# Competency frameworks, nursing perspectives, and interdisciplinary collaborations for good patient care: Delineating boundaries

**DOI:** 10.1111/nup.12402

**Published:** 2022-06-27

**Authors:** Maya Zumstein‐Shaha, Pamela J. Grace

**Affiliations:** ^1^ Department of Health, Master of Science in Nursing Program, Adjunct Head of Program Bern University of Applied Sciences Bern Switzerland; ^2^ Department of Nursing, Faculty for Health University of Witten/Herdecke Witten Germany; ^3^ Boston College William F. Connell School of Nursing Boston Massachusetts USA

**Keywords:** competence frameworks, interprofessional, nursing education, nursing practice

## Abstract

To enhance patient care in the inevitable conditions of complexity that exist in contemporary healthcare, collaboration among healthcare professions is critical. While each profession necessarily has its own primary focus and perspective on the nature of human healthcare needs, these alone are insufficient for meeting the complex needs of patients (and potential patients). Persons are inevitably contextual entities, inseparable from their environments, and are subject to institutional and social barriers that can detract from good care or from accessing healthcare. These are some of the reasons behind current movements to develop competency frameworks that can enhance cross‐disciplinary communication and collaboration. No single profession can claim the big picture. Effective teamwork is essential and requires members of diverse professions to understand the nature of each other's knowledge, skills, roles, perspectives, and perceived responsibilities so that they are optimally utilized on behalf of patients and their families. Interdisciplinary approaches to care permit different aspects of a person's needs to be addressed seamlessly and facilitate the removal of obstacles by engaging the range of resources exemplified by the different professions. Additionally, collaborative efforts are needed to influence policy changes on behalf of individual and social good and to address root causes of poor health especially as these impact society's most vulnerable. Here, we explore both the benefits and the risks of an uncritical acceptance of competency frameworks as a way to enhance interdisciplinary communication. We highlight the importance of anchoring proposed competency domains in the *reason for being* of a given profession and exemplify one way this has been accomplished for advanced practice nursing. Additionally, we argue that having this mooring, permits integration of the various competencies that both enhances professional moral agency and facilitates interdisciplinary collaboration to further the mutual goals of the healthcare professions on behalf of quality patient care.

## 1 INTRODUCTION

To promote person‐centred care, to counter fragmentation, and to reduce over‐ and undertreatment in any setting where healthcare occurs, it is undisputable that the disciplines need to be able to draw upon one another's knowledge and expertise (Deneckere et al., 2013; Dulay et al., 2020; McHugh et al., 2020). The ability to communicate effectively across disciplines is critical for optimal care of individuals and society (Englander & Carraccio, 2018; Grace & Perry, 2013). Thus, members of each profession whether collaborating, consulting, or working as a team, should have a deep understanding of their own role and contributions and be knowledgeable about what the other profession or professions contribute. As a point of clarity, we take “discipline” to mean the knowledge‐base and ‐development aspects of a service‐providing entity such as nursing and medicine, and “profession” to be the knowledgeable and informed practices of said service‐providing entity. Cross‐communication is essential for good patient care and good healthcare more generally. The time for hierarchies has passed. When one group dominates an interdisciplinary conversation, important dimensions of individual and socially responsible care are liable to be lost (Grace et al., 2007).

Ostensibly to address the problem of miscommunication (Englander et al., 2013), the use of competency‐based education has been proposed as capable of improving patient care by the apt preparation of clinicians who can collaborate effectively across disciplines. Various frameworks have been developed and initiated depending on country and profession. The most prominent currently, across countries, was developed for the purpose of directing medical curricula (Batt et al., 2020). While the nursing profession for several decades and in a variety of countries has used the concept of competencies as a way to evaluate clinical skills, the shift to using competency frameworks to underpin the whole curriculum is new and its effects on practice unknown. This, and the fact that proposed frameworks originated in medicine for the purpose of improving medical education is the crux of our concern.

As Batt et al. (2020) note from their scoping review, there is great variation among the methodologies underpinning framework development and “a lack of guidance on how to identify the most appropriate methods of development” (p. 930). Moreover, they found that “existing guidance (for developing competency frameworks) acknowledges that what we consider fit for one setting or profession and intended use may not be for another, hence the flexibility and variability” (p. 931). To date, it remains unclear how or whether the various healthcare professions can both maintain their particular perspective and communicate effectively with each other using competency frameworks, especially when these were developed in and for another discipline. There is concern that these frameworks will develop an ‘authority’ beyond that which is warranted by the evidence of effectiveness for the goal of the specific profession (Batt et al., 2020).

This article aims at providing a critical and cautionary lens with which to review the scope, usefulness, and limits of competency frameworks related both to interdisciplinary communication and professional education. Our concern as experienced nursing clinicians, scholars and educators is specifically for the future of nursing as a distinct (if evolving) profession, whether it is susceptible to absorption into another body, and what losing nursing's focus as discussed shortly, might mean for individuals and society. However, we include a broader exploration of competency frameworks in general and question whether uncritical acceptance might lead to constricted vision and agency within other healthcare professions.

### 1.1 A Swiss example

An example of the use, or rather misuse, of frameworks for nursing and allied health professions, originating in medicine, is that of the situation in Switzerland. For decades, nursing and nursing education had not been regulated in the law, either by the profession or in general. With fundamental changes in education, a new law regulating nonmedical health professions—nursing among them—was conceptualized and only recently implemented. For the new law, both the Bachelor and Master levels of education in nursing and other nonmedical health professions were reviewed (between 2010 and 2017) in terms of learning outcomes and competencies. For this purpose, the competencies of all these professions were required to comply with the CanMEDS framework (2017). This framework was developed by the Royal College of Physicians and Surgeons (RCPSC) in Canada and “is a framework that identifies and describes the abilities physicians require to effectively meet the health care needs of the people they serve” (2022). Grounding the competencies on the CanMEDS framework was stipulated by the Federal Office of Public Health in Switzerland, spearheading the development of this law (Schirlo & Steurer, 2018). In the course of our critique, we argue why this sort of directive is problematic for individuals and society. Such directives narrow the ability of the nursing and healthcare professions to base curricula development, and thus scope and purposes of practice, on goals and perspectives to those that fall under the medical perspective.

### 1.2 General problems with competency frameworks

Subsequently, we highlight possible risks/benefits from the use of competency frameworks as guides in the development of any healthcare profession. But our special concern is developing nurses who can practice well (ethically). Practicing ethically, as we describe later, means using clinical and ethical decision‐making for the benefit of a patient or group or to minimize likely harms. It includes knowing know how to overcome barriers to good practice that arise from patient circumstances, healthcare settings and/or sociopolitical forces. This inevitably requires motivation and an ability to collaborate as well as access to appropriate resources and supports. While much has been written about the benefits of competency frameworks for directing nursing education and nursing work in general, and for improving interdisciplinary collaboration and communication (Englander et al., 2013), the supportive evidence remains scarce (Batt et al., 2020). However, our intention in this manuscript is NOT an outright dismissal of the utility or instrumental nature of certain aspects of competency frameworks but rather to caution against adopting them wholeheartedly without understanding and accounting for their limits and risks. In relation to directing nursing education and future disciplinary knowledge development, competency frameworks may have a place, provided their limits are recognized, and certain modifications are made. In the process of our critique, we explore the concepts of profession, competence, and the history behind the emergence of competencies and competence frameworks and their acceptance in the healthcare lexicon.

### 1.3 Contested nature of the concept of professions

We do use the convention of referring to different practice bodies (medicine, nursing, pharmacy, social work, etc.) as professions; while acknowledging the contested nature of this term, its meaning and purpose (Namazi, 2018; Porter, 1992). Rather than discussing what constitutes a profession and why and to allow us to distinguish among practice entities easily, we refer to them as professions. Thus, core aspects of the idea of professions as generally understood and described in the extant and historical literature (Grace, 1998; Windt, 1989) can be highlighted. Along with Windt (1989), we take the various healthcare service groups/professions to be providing critical services related to human functioning and flourishing without which many healthcare needs would not be met, and healthcare policies would be even more ill‐informed than they sometimes are at present. We also argue that maintaining a focus on fulfilling the human service goals of professions can help free them from the bureaucratic and self‐protective aspects that can hide‐bound them and compound the obstacles to providing good healthcare.

## 2 CONTEMPORARY HEALTHCARE: RELATIONSHIPS WITH COMPETENCY FRAMEWORKS

A contemporary problem for the existence of any of the healthcare professions is the constriction of autonomy that comes with the current incursion of business interests into healthcare systems; whether a particular country's system is mostly privately funded via insurance or mostly publicly funded by taxes. Business interests tend to shift the focus of services from being primarily about furthering the human good of health, to expediency, economy of action, cost‐effectiveness and in some cases maximizing profits. Note, we are not arguing that cost‐effectiveness is unimportant; it is obviously important as a justice issue. Rather, we posit that it should not be the primary concern when human health is key. Managerialism is the term attributed to the use of business principles to organize the functioning of all aspects of an institution (broadly defined) including those who work within it. As summarized by Carlisle (2011) in her critique of the incursion of managerialism into healthcare contexts in the United Kingdom, “managerialism is a hierarchical top‐down command and control style of management concerned with efficiency and control” (p. 286) not human good per se. Managerialist priorities tend not to be as concerned with upstream problems and addressing root causes of ill health that are embedded in societal injustices. Critical service professions are needed to continue to focus on those societal injustices that lead to health disparities. It is also necessary to understand how to overcome managerialist priorities when these work against the health and wellbeing of those to whom, and for whom, the service is provided. Competency frameworks should be able to prepare professionals who can both practice ethically in contemporary environments and question in what ways they may be constrained by managerial directives as well as perhaps from self‐protective or self‐promotive urges of a profession.

Despite external constraints, nursing as a professional group still exists in many countries and its aspirational promises to society are expounded in contemporary codes of ethics (American Nurses Association, 2015; International Council of Nurses., 2012). These codes of ethics have emerged from historical and contemporary theorizing about what is the human ‘good’ nursing exists to provide. In other words, what unmet needs in society does nursing address that are not met by others. In this sense, the service is moral in nature as long as there is some capacity for autonomous action, self‐rule, and policy influence. Moral practice in this sense does not denote an immutable ideal—this is the right action, and this is how to achieve it—but rather an ability to continue to provide a good in evolving circumstances. In healthcare settings we do not have to answer the question of what is the ultimate good (a metaethical question) rather we address the idea of health as a human good, that permits human flourishing and functioning (Meehan, 2003, 2012).

We also acknowledge that the level of autonomy granted any such profession depends, to some extent, on the power of the group to overcome practice obstacles within practice settings or is engendered or limited by the social fabric and sociopolitical influences within societies. We argue, for example, that stipulating, as the Swiss law does, that nursing education be based on medically grounded competency frameworks, will result in inevitable limitations on curricula. In turn there will be potentially detrimental effects on nursing, nursing education and the development of ethically effective nurses; nurses who can practice well from the nursing perspective of humanizing the healthcare environment (Willis et al., 2008).

### 2.1 Interprofessional collaboration and competency frameworks

Next, we discuss and exemplify nursing's role and perspective within the context of interprofessional healthcare teamwork in institutions such as hospitals and clinics and relate this the aspirations of competency frameworks. Outside of institutional settings, nurses also depend on other disciplines for knowledge or skills that are outside of their realm of expertise. The core of interprofessional healthcare teams in hospital and clinic settings tends to include professionals from medicine and nursing. However, many other disciplines may be members of an interprofessional team depending on setting, circumstances, and desirable expertise.

Interprofessional teamwork, though, remains challenging. As described by Zajac et al. (2021), “challenges that face healthcare teams relate to accountability, conflict management, decision‐making, reflecting on progress, and coaching” (Abstract, para 3). Current professional education and communication are noted as an important barrier to effective interprofessional teamwork (Dulay et al., 2020; Olson & Graber, 2020; Petri, 2010). It has been found that the students from their respective disciplines and professions sometimes neither quite understand one another, nor have a good grasp on the educational background and levels of skills and competencies of one another. Misconceptions may occur, or misinterpretations may lead to suboptimal care of a patient and even grave outcomes (Fukkink & Lalihatu, 2020; van der Gulden et al., 2020).

Growing recognition of barriers to effective teamwork, along with a contemporary paradigm shift in healthcare education from ‘structure and process models’ to ‘competency frameworks’ as discussed later, led to the development of sets of competencies presumed intelligible to members of all healthcare professions and to adequately capture what is needed for good healthcare (Englander et al., 2013). Significantly, we question whether framework grounded in one profession provide enough depth and breadth for the good health of individuals and society. As noted in the Swiss example, building laws on the CanMEDS framework alone is liable to constrict the spheres of concern related to individual and population health. As the CanMEDS and Englander et al. (2013) frameworks have gained traction in healthcare professional education, in a way that is seemingly irreversible at this point, the main purpose of this paper is to argue that—for any given profession—competencies proposed as essential for clinicians should be anchored in its reason for existence as a separate entity. Without such an anchor there is likely to be a drift toward accepting the perspective of the hierarchically dominant group (Herman, 2017; Tibbetts et al., 2022) and loss of willingness to question the status quo on the part of a profession's scholars and researchers

Thus paradoxically, instead of providing for communication among the professions that facilitates a multidimensional approach to good care of persons, a universal competency framework is liable to cause dissolution of the power of professions as the emphasis shifts to achieving outcomes desired by stakeholders who may or may not have ‘human good’ as a bottom line. Alternatively, the perspectives of the more powerful discipline on what constitutes good practice may hold sway. A better understanding of the role and limits of competency frameworks might allow us to harness their power for good and circumscribe their boundaries.

### 2.2 Competence and competency: Meaning and conceptual limits

There is no single origin for the competency movement, nor do concepts of competence and competency have a stable explanatory definition (Gervais, 2016). One reason for this is a lack of sound theoretical underpinnings (Hodge, 2007) that apply across the continuum of educational levels and purposes.

Ideas about competency as an attribute of human beings can be traced back to medieval trades and guilds. Skills were learned “by working with a master” and apprentices “were awarded credentials” upon having reached a certain standard (Horton, 2000; pp. 306–307). Later during the Industrial Revolution, an interest in people having appropriate skills for a technical job resulted in standardized training tailored to the necessary functions. In the last several decades, the competency movement has flourished in response to perspectives on economic competition among countries (Horton, 2000). Equally, the belief that better educated people perform their work more efficiently and better, was influential, thus giving the respective countries a trade advantage (Avis, 2000; Horton, 2000). Various factions, in both business and education, have been persuaded that the development of competencies is a superior way to develop skills and abilities in people. While there may be some validity to such arguments when describing general education or even for developing discrete skills and ability, it is not so clear that a focus on advancing skills and abilities is sufficient to develop human service professionals. Critical human services are those that facilitate human flourishing in some way, such as education, healthcare and law, among others.

Associated with the problem of differing ultimate aims between business and human service professions, is the problem of defining the concepts of competence and competencies. The meanings of these terms, even when some consensus exists, vary based on purpose and setting. Some commentators have urged that we account for distinction in meaning between competence and competencies (Norris, 1991). In education settings, the former denotes an overall condition or ability, “which refers to what an individual knows and can do in a subject area” or situation (Messick, 1984; p. 217). It is maintained that individuals obtain knowledge through “instruction or experience” or a combination of these (Messick, 1984; p. 217). Thus, knowledge is demonstrative of the ability of integration of different competencies and is more than the sum of its parts.

Competencies, in contrast, are more discrete behavioural abilities to complete a particular task. For healthcare professionals, demonstrated performance of multiple competencies is supposed as necessary for good practice. Competencies may vary in sophistication depending on the level of learner and beginner to expert practice expectations (AACN, 2021). However, accomplishing a list of competencies, regardless of level of complexity, while perhaps necessary, is not sufficient for good practice as nursing moral agency is not reducible to a simple gesture or activity. Neither is integrated practice competence alone sufficient to analyze and overcome contextual barriers to practice, in the absence of abilities and motivation to critically evaluate conflictual situations or adopted views. Thus, both overall competence for a job, position or effective management of a patient's situation and competency‐for‐a‐particular‐task are abstract and fluid concepts, understood differently, depending on who is defining them, for which purpose, and how achievement will be evaluated. Additionally, there are different levels of competence or competency, basic to advanced. Perhaps, most importantly for both is that ‘measurable outcomes’ of some sorts delineate the achievement of the competency, regardless of whether that competency is basic, more advanced or integrated in overall competent practice. As a simple concrete example of measurable outcome for a task, a basic competency skill related to evaluating blood pressure might be demonstrated by efficient, consistent, and accurate measurement of blood pressure across different aged patients with different morbidities. Achieving a more advanced competency might be demonstrated by an ability to relate the blood pressure measurement to the person's overall health status. The latter achievement is representative of the overall competence/competency of a nurse related to the meaning of the blood pressure measurement in the context of the person's condition and contextual nuances followed by appropriate actions (Posadas‐Collado et al., 2022). However, root causes of illness such as result from uncontrolled hypertension often derive from disadvantage, lack of access or resources require more sophisticated characteristics for their address. Thus, the notions of ‘competence’ and ‘competency’ are pre‐defined by what experts and other stakeholders determine is a desired skill or ability.

### 2.3 Competence‐based learning

Horton (2000), a public sector scholar, traced the history of competency‐based learning (CBL) as it developed from managerial ideals and managerialism. Not surprisingly, an emerging emphasis on CBL, coincides with the proliferation of modern business schools in the mid‐1900s (Melo & Beck, 2014). Managerialism is linked to competency‐based learning and ideologically underpins it (Avis, 2000), in that it is about achieving visible or measurable outcomes. Managerial objectives have carried over to the education sector in many countries as governments seek to standardize education for the purpose of developing efficient human capital. Note ‘efficient human capital’ is a business, not a human services term. Business and human services necessarily differ in aims. One is ultimately aimed at an economic bottom‐line and the other a human good. Therefore, potential outcomes from educational frameworks based on such premises may not include philosophical critique and ethical analysis. For better understanding of reasons for nursing to refer to such educational frameworks, it is helpful to explore whether there are meaningful differences between competence and competencies as some have proposed (Norris, 1991), and if so whether such differences should be accounted for when using competency frameworks.

In the 1970s, there was some controversy about the difference between branches of the competency movement that were “performance based” versus those that were “competence based” in relation to the education of teachers. Some argued that ‘performance” focuses on evaluating observable skills and cannot capture the nuances of professional knowledge; thus, they preferred the term “competency” as being broader (Norton et al., 1978). We critique later that while the second term is preferable if it is indeed capable of accounting for the not so easily measurable knowledge that healthcare professionals possess, it may not capture the characteristics needed for good nursing practice as this inevitably occurs within the complexity of contemporary healthcare settings. In current healthcare settings, a multitude of obstructions to good care exist, including but not limited to the economic circumstances of the institution or setting. Currently, the effects of the COVID‐19 pandemic are causing nurse compassion fatigue and nurse attrition (Alharbi et al., 2020). Possession of desirable practice competencies (however these are determined) will not be sufficient to overcome such obstacles to good practice. The discipline of medicine has also realized how such crises such as COVID‐19 can interfere with the process of developing competences (Ryan et al., 2021). Thus, when competency frameworks for nursing education are adopted as the exclusive organizing tools, they are likely to fall short of producing nurses who consistently practice ethically (well) and are equipped with the political skills to overcome practice problems. Practice problems may be caused by persisting professional hierarchies, social or institutional foci on economic expediencies over human good, or other issues.

Hodge (2007) critiques that Competency Based Training (CBT), as the movement is called in Australia, does not derive from a particular theoretical perspective but rather is “an amalgam of separate theoretical components alloyed in the crucible of powerful political forces, and that responsiveness to social and cultural pressures remains a significant feature of CBT” (p. 180). In other words, contemporary ideas about competency tend to be of political and/or economic origin and goals, rather than being based in a theoretical foundation in human flourishing. Thus, competency‐based educational frameworks for nursing curricula, while having some merit in terms of providing for consistency in knowledge and skill acquisition in specified or specifiable circumstances, fall short of developing “good” or expert practitioners. They cannot help us answer the questions: “for what reasons are we developing these competencies, which competencies are most likely to help us achieve professional goals, what evaluation methods are sound?”

Theoretically derived goals and perspectives of the profession are better anchors for nursing education. Importantly nurse philosophers and theorists, while having theoretical differences, have cohesively argued that nursing is concerned with the health of persons viewed as contextual beings whose health status is at least partly subjective. The role of nurses, then, is to account for contextual, meaning‐related, psychological, and physical aspects in addressing individual health needs (Willis et al., 2008). Though the goals of the healthcare professions may be mutually held, having a focus on restoring and promoting health, the way to achieve these goals varies on the theoretically derived perspectives of each profession. Reliance on the development of competencies, then, would be instrumental and require additional abilities such as philosophical critique and ethical analysis for achieving theoretically and ethically derived professional goals (Grace & Perry, 2013). Philosophical critique is needed to determine, which competencies are important, what is needed to develop these, how to evaluate them, and how they fit within nursing's philosophical and theoretical endeavours. Similarly, ethical analysis is necessary to determine ethically adequate care and to identify threats to good nursing care (Grace & Milliken, 2016). Ethically adequate care would be that which optimizes health for an individual (and in turn society) and permits them to gain or regain a sense of integrity physically, psychologically and/or socially. Additionally, an understanding of the profession's ethical responsibilities and how to frame this within the language of ethics and human rights is one way to facilitate interdisciplinary work. It permits the nursing perspective on a patient care situation to be articulated when ethical conflicts and dilemmas emerge (Grace, 2018). Importantly, understanding both the potential benefits and pitfalls of competency models of education permits the qualified rather than unconditional use of them. It remains to be seen whether shared competencies among the healthcare professions can or do overcome communication and collaborative difficulties caused by historical hierarchies and the influence of managerial or business principles.

Competencies regardless of definition are about capabilities to engage in those actions deemed by the designers of curricular competency frameworks as preferable skills, knowledge, and actions for specific purposes. But more is needed to determine what preferable actions are when this is not clear. Preferable actions in conflictual situations require a questioning of the situation, exposure of underlying assumptions and root causes, including sociopolitical movements that disadvantage some in comparison to others. There is a danger that loss of emphasis on the obligation to challenge accepted practices and injustices—a potential side‐effect of competency curricula frameworks—that arise from within healthcare systems and societal circumstances, will weaken the profession and leave it even more vulnerable to influences that do not have human good as the primary objective. Ultimately, it is the individuals in need of nursing and healthcare services who will suffer the most.

Competency frameworks, because they tend to prioritize the needs of institutional stakeholders for efficiency and economy over the needs of patients, remain reductionistic (Reeves et al., 2009), and not facilitative of nurse moral agency, regardless of broader intentions such as those envisioned by the AACN (*American Association of Colleges of Nursing*) in its preamble to the 2021 *Essentials*. However, educational curricula need to develop nurses who will question how a given competency facilitates patient good from a nursing perspective.

## 3 COMPETENCY FRAMEWORKS AND NURSE MORAL AGENCY

Elsewhere, nurses' abilities to consistently practice well and in accord with professional ethical responsibilities despite obstacle has been termed nurse moral agency (Grace, 2018; Grace & Milliken, 2016; Robinson et al., 2014). In brief, nurse moral agency is the motivation, knowledge, and skills to achieve patient “good” in everyday work and the capacity to work to overcome barriers to achievement of that good. Patient “good” in this sense is informed by contextual circumstances. For example, a person with stage IV cancer does not want to continue treatments, but the family along with the oncologist are pressuring her to continue. The nurse understands that the treatments, while possibly prolonging the person's life, might also prolong her suffering. And the patient has provided good reasons why she does not want more chemotherapy. However, the oncologist values “success” but does not consider the patient's idea of what this might be, until apprised by the nurse. The oncologist is listening to the family's desires over those of the patient. The good action in this case is to support the patient to get what she wants and the family to deal with their anticipated grieving.

Understanding how and why the nursing profession in many countries has come to accept “competency frameworks” as appropriate structures for nursing education and potentially nursing knowledge development, is important. We argue, these should be seen as instruments to develop knowledge and skills and facilitate collaboration, while being anchored in nursing's goals and perspectives on how those goals are best met (Author, In Press). More evidence is needed about the risks and benefits of competency frameworks when used to direct curricula, best practices in developing competencies (Batt et al., 2020) and so on. Thus, we see the idea of developing competencies as possibly helpful for interdisciplinary communication but inadequate for developing nurses who can exercise their moral agency even when faced with adverse conditions. As noted later, the development of competency frameworks that are visibly and unmistakably rooted in the profession's theoretical/philosophical perspectives and goals, and which allow for critique of policies and proposed standards, might be able to overcome most, if not all, critiques.

A problem raised by Batt et al. (2020), in their extensive review of competence frameworks and their development, is that what is seen as a desired end product may not be well supported by evidence or might be given priority over what is actually a good practice in the circumstances. It follows, then, that how the terms are defined, what evidence exists for relying on them, and for what purpose they are to be used is critical to understanding the respective demands, subsequent operationalization, and limitations. One definition proffered in the healthcare arena that does not add much specificity, is that competencies encompass a “complex set of behaviours built on the components of knowledge, skills, attitudes, and ‘competence’ as personal ability” (Carraccio et al., 2002; p. 362). In essence, a reliable measure of the desired competency depends both on the use of sound evaluation tools or skilled observations and the value of the competency in providing a “good” (the reason that healthcare profession exists). Moreover, “the competencies drafted reflect their particular views of what constitutes ‘best practice’ at the point in time they were created” (Reeves et al., 2009; p. 452).

The paradigm shift in healthcare education to a competency‐based curricula, arguably, began with medicine. A perceived growing divide between medical education and clinical realities in conjunction with rapid developments and advances in the medical field led to a review of medical curricula in many countries as they were seen as inadequate for modern medical practice. To overcome these discrepancies, medical school curricula in the United States first shifted to a “structure and process” design (Carraccio et al., 2002). Structure and process models of curricula emphasized the delivery of desired content within a set period of time, after which the student is eligible to graduate. For practice professions, developing certain skills and competencies were expectations within the broader curriculum and were based on developing the effective clinician, one who could identify and meet a patient's needs based on disciplinary goals. This person is what Bain (2018) calls the good or ethical physician and expectation of whom is continued professional development in the interests of patients.

In structure and process models the teacher is in charge of motivating learning and the student's knowledge acquisition level is evaluated against that of their peers. The end‐result of the period of study is a novice professional. In contrast, competency models focus on students’ development of discrete knowledge and skills related to application in practice. Students and teachers both have a (nonhierarchical) role in the development of the competency over a variable time‐period (Carraccio et al., 2002). Acquisition of the competency as ready for the practice setting, then, can be measured. Note that a distinction needs to be drawn between a competency or specific skill that is acquired and competence in the practice role, which is about the overall ability of a person to practice well. Some commentators have noted that there are gradations of competency from basic to adept (Fukada, 2018; Watson, 2002), that depend to a certain extent on education level, experience, and motivation to continue acquiring knowledge.

Nursing education in many countries has for several decades included testing of student nurse competencies for various basic skills such as health assessment and procedures. These competencies were seen as instrumental to the overall goal of enabling good practice from a nursing perspective that nurses are complex beings and that good nursing care accounts for individual differences, including contextual and environmental influences (Cowan et al., 2005; Scott Tilley, 2008; Taylor, 1995). The whole nursing curriculum, however, has not until now been based in the idea of developing competencies. Indeed Benner's (2001) research highlighted that time in practice is needed for a person to first develop competence and then expertise. She notes that knowledge is “imbedded in actual nursing practice… it accrues over time” (p. 1). Evidence is lacking currently (perhaps due to the novelty of competence frameworks) about how effective, and in what ways, competency frameworks are in preparing nurses (and other professionals) to meet their profession's goals. Our argument centres on the idea that nursing goals and perspectives should anchor and provide coherence to any individual set of competencies. Anchoring them this way permits the relationship among sets of competencies to be visible and complementary, but it also allows for critique and remedy of environments that hinder good patient care. As an example, competency caring for a postsurgical patient would mean the ability to anticipate and act to remediate a range of postoperative complications, but nursing perspectives would ensure the person's individual needs for reassurance, family presence and so on were also met. Additionally, when nurses understand their roles, perspectives, and responsibilities they are more likely to engage in interdisciplinary discourse in a more confident way (Lee et al., 2020).

To reiterate, our discussion demonstrates that we can accept competency frameworks as helpful education tools without agreeing that they are sufficient for consistently good practice in the face of barriers to meeting the profession's goals, nor that as it stands that they improve interdisciplinary communication. In particular, competency frameworks seem to focus predominantly on activities instead of more complex, less easily measurable attitudes (Batt et al., 2020), or motivations to act, which are both necessary for nursing moral agency as discussed in more detail earlier. From here on, we use the terms ‘competence’ or ‘competency’ synonymously to denote task‐oriented, situational, or more global achievements, specifying as necessary which sense we are using. Our main concern is to ensure ethical interests in critiquing the sometimes “restrictive fit” of competencies, are not blocked by the demands of pre‐set competencies and their measurement criteria. Leaders and educators of the medical profession in various countries have also recognized some of the limitations of competence frameworks and have suggested the concept of *entrustable professional activities* (EPA) as a way to circumvent some of these concerns. However, EPAs are subject to their own critiques that they are not able to consistently do what is expected of them (Krupat, 2018).

### 3.1 Competence frameworks and entrustable professional activities

As Norris (1991) notes of the earlier years of the competence movement into healthcare education among other practices, despite critiques, “(E)verybody is talking about competence. It is an El Dorado of a word with a wealth of meanings and the appropriate connotations for utilitarian times” (p. 331). Competency frameworks aim for a certain level of standardization of practices, and assurance that practitioners can perform their work well. Currently it is expected that educational competency frameworks can standardize the basics of what someone needs to know to act to complete a task or set of interrelated tasks or resolve a more complex problem. The aim of these tasks is either objectively preset based on evidence or more subjectively preset based on a combination of evidence (where this exists and is sound) and the deliberations of experts. In standardizing approaches to action there are several concerns for the nursing profession (as well as for other human service professions). Perhaps the biggest concern is the potential loss of focus on developing practitioners who understand and can meet the philosophically developed and described goals and purposes of nursing, and when obstructed from doing so will find ways to surmount the obstacles. That is, there is a danger that the development of clinician moral agency, that is the development of certain characteristics (professional formation) that motivate intentional action to bring about the needed healthcare good for a patient or group, will be neglected.

Medicine's remedy for the critique that focusing on developing practitioner competencies tends to compartmentalize rather than integrate patient care is the addition of entrustable professional activities (EPAs). However, EPAs have received their own criticisms consisting in the argument that they are not capable of remedying the problem for which they are proposed as a solution (Krupat, 2018).

EPAs represent “a pragmatic list of activities—a core set of tasks or responsibilities that all (medical) residents could be trusted to do independently by the time they (have) completed their training” (Krupat, 2018; p. 371). Cate and Scheele (2007) argued that the addition of EPAs to competency frameworks provide a bridge from achievement of certain competencies or sets of competencies to their enactment in practice. In essence, EPA are those responsibilities that faculty trust a medical student or resident to carry out unsupervised, having achieved the appropriate set of related competencies and successfully demonstrated effective patient care in a discrete circumstance (Cate, 2013; p. 691).

Krupat (2018), however, argues that neither competencies nor the addition of EPA in medical education seem capable of achieving their aims, and states: “that we are measuring the wrong things, measuring the measurable rather than the important” (p. 371). They foreground skills but distance aspects of professional presence such as “the ability to communicate with patients, and the ability to deliver quality patient care regardless of race/ethnicity, gender, or sexual orientation” (p. 372). Others raise issues of measurement and method, noting problems in making valid and reliable Assessments. For example, Lurie et al. (2009) conducted a systematic review of the literature to explore how sound the measurements of the US Accreditation Council for Graduate Medical Education's (ACGME) six general competencies were. They discovered that “quantitative/psychometric studies of evaluation tools failed to develop measures reflecting the six competencies in a reliable or valid way” (p. 301). Krupat's (2018) critique is that despite medical educators’ attempts to overcome criticism that competency frameworks can be reductionistic by augmenting them with the concept of core EPAs, problems of conceptualizing what competencies are needed and how to measure these across levels of activity complexity, remain.

Nursing has, as noted earlier, for the past several decades used acquisition of discreet task‐oriented competencies as part of a more “structure and process” type curriculum designed to develop nurses who can practice from a nursing perspective. Although in the US there are curricula variations depending on the route to licensing. For example, pre‐licensing education could be 2 years for an associate degree, 4 years for a baccalaureate, and 3 years for hospital apprentice type programmes. Nevertheless, a patient‐centred rather than disease‐centred philosophy prevailed in accord with philosophical and theoretical understandings of the role of nurses. Across countries as evidenced in the literature, nursing curricula are based on philosophical and theoretical underpinnings of nursing as these have developed over time and in response to societal needs and practice environments.

So, the idea of achieving competency to perform tasks is not new. Contemporary criticisms in the United States and elsewhere of the adequacy of nursing education include that nurses are not always well prepared for the realities of practice nor for their ethical responsibilities to patients in spite of educational efforts to promote nurse moral agency (Jurchak et al., 2017). However, the paradigm shift underway currently involves a transition to competency frameworks for the whole curricula (AACN, 2021). The problems faced in the design of medical curricula, using competency frameworks should give the nursing profession pause. A question for nursing and nursing education to resolve, then, is given the existing critiques of competency frameworks in medical education even with the addition of EPAs, ‘what are the dangers of uncritically following the lead of medical education in the turn to using competency frameworks to direct curricula’? How can we ensure that competency frameworks prepare nurses to practice well, overcome obstacles to good patient care, and account for root causes of ill health that stem from social arrangements and structural injustices?

### 3.2 Structure and process models versus competency models

It was hoped that a shift to defining required competencies at the outset and using these to shape learning content would ensure that students obtained the knowledge and skills relevant to the subsequent practice. Additionally, there was an expectation that additional and/or deepened learning would be motivated. Competency‐based medical education is focused on the ability to apply knowledge and skills in practice. A competency‐based curriculum specifies measurable outcomes as proof that the student has reached a preset minimum level. Little evaluation has been conducted on the outcomes of competency‐based education. However, competency‐based education is viewed more favourably than previous educational frameworks. It is critical that the competencies are clearly identified and that outcomes are distinct and measurable and faculty themselves are competent educators. In addition, faculty and students/learners need to be motivated to be engaged in the learning process. Similarly, the clinical faculty also needs to be well trained in the competencies to advise students/learners appropriately (Bacchus et al., 2017; Dagnone et al., 2020).

As with any curricula plan, reliability of results depends upon the design, implementation, expertise of faculty—academic or clinical—and whether the evaluation measures accurately capture the student's capacity to use knowledge and skills in practice. A primary concern is that patient needs and patient safety “foreground” the activity. As Sebok‐Syer et al. (2021) note, “EPAs are an approach to designing and implementing CBME (competency‐based medical education), which begins with determining what patients need and then uses that information to develop curricula and approaches to assessment that ensure those needs can be met. Viewing EPAs solely as an assessment framework foregrounds, almost exclusively, the trainee. Casting this spotlight on the trainee may leave the patient in the shadows” (p. S76).

Additionally, Englander et al. (2013) developed a taxonomy that they claim can be used and understood across different health professions although with some modifications for the various professions. They suggest that a set of common competencies would facilitate interdisciplinary access to literature resources available from the various disciplines by articulating a problem in a consistent way. Drawing on a systematic literature review, and a comprehensive set of competencies identified by the Accreditation Council on Graduate Medical Education (ACGME) (2021) and adopted in 1999 in conjunction with the American Board of Medical Specialties Englander's modified framework (2013) was proposed: Patient Care (PC), Medical Knowledge (MK), Interpersonal and Communication Skills (ICS), Professionalism (P), Practice‐Based Learning and Improvement (PBLI), and Systems‐Based Practice (SBP). Each one of these domains already came with a set of competencies (Englander et al., 2013; p. 1089). CanMEDS is another framework, mentioned earlier in conjunction with the legislation in Switzerland of CanMEDS to underpin the law for all nonmedical healthcare professionals. It was developed by the Royal College of Physicians and Surgeons of Canada (RCPSC) in the early 1990s and updated periodically (2022). CanMEDS has been adopted for use in a range of countries including Switzerland, the home base of the first author.

These three frameworks—EPA, CanMEDS, and the ACGME/ABMS framework—have only been implemented recently in medical education. It appears that the most experience has been obtained with the CanMEDS‐framework. Recent studies exploring various aspects of the CanMEDS‐framework in relation to generally understood aspects of the physician's role found some gaps. For Bacchus et al. (2017) competence, context, and conduct are of equal importance in good physician practice. However, they found that context was not represented in the framework and conduct was sorely underrepresented. Other studies suggest that the CanMEDS‐framework may need to be more comprehensive to capture un‐ or underrepresented areas of practice in medicine. Some domains remain as yet open for improvement such as the personal development domain (Bacchus et al., 2017; Dagnone et al., 2020; de Graaf et al., 2021; Renting et al., 2017; Voll & Valiante, 2017).

The EPA‐framework has also been scrutinized for its impact on learning. It has been revealed that the EPA‐framework lacks ways for students to develop ethically sound practice and the continuity of learning is hindered as clinical exposure of medical students happens in blocks distributed across medical education. The piecemeal arrangement is taken as preventing medical students from engaging more fully and consistently with patients and, thus, developing ethically sound practice. This situation is highly undesirable as patient outcomes can suffer as well as student learning outcomes (Englander & Carraccio, 2018; Hong et al., 2021). Eventually, medical students or newly qualified physicians may lack sufficient resources to prevail in medicine and may leave the profession prematurely (Bellini et al., 2019). The problem of ensuring and evaluating competencies has been highlighted recently as a result of practice crises exacerbated by the COVID‐19 pandemic (Holmboe, 2021). Similarly, it has been recommended that clinical mentors and advisors to medical students review their teaching abilities and focus on developing ethical sensibility and sensitivity toward the learning process of the medical students to promote learning and the development of ethically sound practice (Bellini et al., 2019; Busche et al., 2016; Moore et al., 2017). Being more socially responsible to patients and the public is one of the reasons given for the paradigm shift in medical education, thus this is an important area for competency frameworks to address but an extremely difficult one to evaluate. All of these critiques validate our concerns about the possibly constricting nature of competency curricula frameworks for all healthcare professionals.

## 4 NURSING PROFESSION'S REQUIRED COMPETENCIES

As nurse clinicians, scholars, and educators our concerns relate to the role of nursing's philosophical, theoretical, and ethical work and how they are incorporated in competency frameworks. While definitions and understandings of what constitutes “nursing science” vary, a recent suggestion is that it comprises “both the process of inquiry and the accumulating body of contingent truths, that support the historically derived unifying focus of nursing” (Grace & Zumstein‐Shaha, 2020; Section 5). The central unifying focus of nursing described by Willis et al., from a survey of scholarly writings and discussions with nursing scholars involves humanizing the environment and “facilitating meaning, choice, quality of life, and healing in living and dying” (2008, p. E28). The goals of nursing have been developed over time in relation to the particular role of nurses in addressing the unmet needs of society (ICN, 2012). Nursing science is viewed as process and content serves as the basis for refining existing philosophies and theories about the reason for the discipline's existence and conceptualizing new theories as necessary. The purpose of nursing science is to facilitate the profession's advancement as provider of a distinct critical human service (Grace & Zumstein‐Shaha, 2020). As such, nursing is concerned with providing care to persons who have specific needs due to health risks and/or disease situations. The goal of nursing care is to support persons with these specific needs to manage daily life as well as possible and/or to live towards a good death. As noted earlier, nursing and other health care professions have mutual goals related to human wellbeing. However, the fundamental perspectives of the professions differ and each perspective is critical to integrated care of an individual or to providing healthcare services for society. The second author's experiences on ethics committees and institutional review boards exemplify the importance of the voice of nursing in ensuring that the patient's or study participant's experiences remain central to the discussion.

For competencies to be adequate for good nursing practice, they should be anchored in nursing goals and perspectives; while recognizing how the contributions of allied professionals, based on their goals and perspective can complete the picture. Anchoring the desired—by the profession and other stakeholders such as employers – sets of competencies in nursing goals and perspectives permits the development of curricula in a way that the interface and integration of the various sets of competencies are visible and relate to the profession's goals.

The following have all been proposed as necessary to develop good nurses: knowledge of nursing's development as a discipline (knowledge development arm) and profession (practice arm), knowledge from other disciplines refracted through a nursing lens that views persons as inseparable from their contexts and circumstances; an understanding of the inherently ethical nature of nursing practice from the everyday to the dilemmatic (Milliken & Grace, 2017); the development of characteristics that predispose one to act to remedy problems that affect patient care either at the level of the individual or outside the patient care situation as needed (Benner, 2001, 2010; Lee et al., 2020; Paans et al., 2017). Nurses self‐evidently must also be able to collaborate with others to resolve barriers to good care that are beyond their singular ability to resolve. This is public or social expectation of persons who seek health and nursing care and is a fiduciary or trust‐based responsibility. It is also an ethical resonsibility. One cannot be held ethically responsible for actions when there are no choices to be made (the philosophical problem of ‘ought’ implies ‘can’). If the healthcare professions cease to have control over practice or areas of practice, then we need to be transparent to the public that we cannot do what they expect of us and why.

### 4.1 A nursing‐based competency framework exemplar: Advanced practice nursing

In nursing currently, one framework exists as an example of how goals and perspectives of nursing can underpin a curricula and integrate competencies. It can do so in a way that the clinician remains alert to problematic standards, policies and practices that are problematic for those in need of healthcare. These competencies are designed with the idea that the need for interdisciplinary collaborations is inevitable in the contemporary complexity of healthcare environments. This framework is grounded in the concept of Advanced Practice Nursing, which aimed at providing a sound nursing basis for all Advanced Nursing Practice roles about to emerge. Thus, the concept of Advanced Practice Nursing (APN) was developed to appropriately prepare nurses to assume such roles in clinical practice (Tracy & O'Grady, 2019). Respectively, curricula were organized to culminate in a graduate nursing degree such as a Master of Science in Nursing. The central competency is a sound clinical practice based on direct clinical practice experiences. The sub‐competencies are together designed to facilitate good clinical practice and include: guidance and coaching, consultation, evidence‐based practice, leadership, collaboration, and ethical decision making. The central competency of direct clinical practice indicates that the competent nurse professional is actively working in patient care from a nursing perspective even when engaging in more advanced skills than required of a bedside or point‐of‐care nurse. They need to demonstrate expertise in patient education and support and this requires understanding the particular needs of individuals. Among nursing colleagues, advanced practice nurses are reference persons, providing important information such as introducing the latest standard of care and questioning those that do not serve patients well. Hence, these advanced practice nurses possess the latest knowledge of specific issues and educate their fellow nurses and other healthcare professionals.

An expectation of advanced practice nurses is that they can identify knowledge or evidence voids and inadequate or poor practice environments. Ideally, they contribute to the development of new knowledge. By pointing out gaps in the literature or trying to integrate innovation, these APNs demonstrate leadership. Often in advance practice settings, there is an interprofessional healthcare team or there are interprofessional collaborations including referals. Hence, APNs are expected to demonstrate capabilities of promoting and maintaining interprofessional collaboration. Finally, APNs are expected to identify ethically problematic situations and be engaged with others in exploring and trying to resolve morally conflictual events. This competency framework has been established worldwide for the implementation of advanced practice nurses. However, it is not necessarily used in this form for undergraduate nurses as the framework focuses on nurses with post‐graduate degrees. As such, the framework does not inform the same level of education in nursing as the previously introduced frameworks, that is, the EPA, CanMEDs, and the ACGME/ABMS. Nevertheless, the concept of Advanced Nursing Practice is interesting as it is moored in the nursing discipline and as such is coherent with its core ideas and reason for being a profession (Gaidys, 2011). While we have this as an example, more data needs to be gathered to demonstrate effectiveness in maintaining the nursing focus on human wellbeing and nurse moral agency.

## 5 ADEQUACY OF MEDICAL EDUCATION FRAMEWORKS FOR THE NURSING PROFESSION

Contemporary nursing takes place in increasingly complex sociopolitical, biotechnological, and economic environments. Practicing well—which we equate with practicing ethically—requires a firm understanding of the goals and perspectives of the profession as these evolved over time (Grace, 2001, 2018). A broad knowledge base is also required to address healthcare needs from the nursing perspective of humanizing healthcare environments, particularizing care to the individual, and promoting health for groups and society. More specific and in‐depth knowledge is also requisite to providing care for particular groups or those with specialized needs. Clinicians such as physicians and nurses are viewed to be in the best situation to address the health needs of a person given an objective evaluation of that person's physical or psychological condition mapped to existing knowledge and in concert with the patient's subjective experience of his or her situation, culture, values, and preferences. Patients may often know what they want but do not necessarily know the (best) path to achievement. To some extent, they are dependent on health professionals. Patients entrust their needs and wishes regarding their health risk or disease, assuming that health professionals keep patients’ best interests as a primary concern. This is an important point in view of the interprofessional team acting to provide care, and constraints imposed by the environment or system within which the services are delivered.

Above all, as we have argued nurses need to be prepared as moral agents capable of addressing, and motivated to address, for example, conflictual situations that hinder their ability to utilize their knowledge and practice well. Among the problems faced are the constraints of the workplace. Both an understanding of the scope of professional responsibility and the ability to engage in ethical decision‐making are the purview of ethics education (Avci, 2017; Benner, 2010; Cannaerts et al., 2014; Grace & Milliken, 2016). An adequate ethics education is integral to consistently good practice, which should be a priority for the profession but increasingly it is not. There are many reasons for this including: anecdotally—from our experiences in academia—ethics is seen by nursing faculty as a rather esoteric subject; it is too difficult to pack ethics into already crowded curricula based on developing clinical competence; the needs of stakeholders such as hospital and clinic administrators take precedent over professional values; politically motivated trainings leading to health professionals without a nursing background but delivering basic nursing care; nursing faculty themselves lack the necessary knowledge to incorporate ethics into their work. A better understanding of the integral nature of ethics to good practice is urgently needed (Grace, 2018). The integral nature of ethics to nursing goals and perspectives and good practice was in danger of becoming de‐emphasized in the new AACN (2021) competency‐based curricula—however, nursing ethics scholars designed a framework to provide educators with resources to integrate ethics across the competency domains and this is now available.

### 5.1 Medical and nursing education: Connections and disconnections

Since the inception of modern‐day nursing education, not surprisingly and despite different practice perspectives a close connection with medical education can be observed. Therefore, we focused our critique on the usefulness and limits of competency frameworks derived from medicine for structuring nursing education. Specifically, we were concerned with the CanMEDS, or CanMEDS in conjunction with EPA framework, and the Englander and colleagues' set of competencies (Carraccio et al., 2002; p. 361; Kazevman et al., 2021; Renting et al., 2017). These frameworks have received their own critiques related to medical curricula and their ability to develop physicians who can practice well and meet their professional goals. However, we had additional concerns that competency frameworks were not fit for the purpose of nursing education without major modifications and awareness of their limits. Our concern is that without an anchor in the profession and without further evidence of their efficacy for good patient care they may not be capable of either serving individual patients well or addressing the root causes of ill health in societal conditions and disparities. In available competency frameworks for nursing education, nursing goals and perspectives are for the most part invisible. For example, the preamble to the new AACN *Essentials* (2021) document does acknowledge the importance of nursing's theoretical and ethical underpinnings for the development of the profession, but it is far from clear how these guided the choice of competency domains or how these interrelate. Lacking in them is a matrix from which an interrelated set of encompassing competencies can form a coherent curriculum that focuses on more than task accomplishment, but rather on meeting the needs of individual patients viewed as inherently contextual beings and influencing policies that do not serve the population well.

Further, there is a danger that competencies become reductionistic (Reeves et al., 2009) and distract nurses from critiquing the status quo of environmental and other obstacles to the exercise of the newly gained competencies. Such less than optimal outcomes have been observed in medical education (Aoun et al., 2019; Busche et al., 2016; Giuliani et al., 2020). EPAs are new to nursing and would need to be adapted. There appears to be some merit in utilizing EPA in nursing to support evidence‐based practice education and transfer into practice (Lau et al., 2020; Leung et al., 2016; Wagner et al., 2018) but evaluating these requires expert faculty.

Achieving “competency” in certain actions without thinking about whether the action needs to be modified to a particular person's needs is problematic for nurses. Indeed, this tension between ensuring certain general minimums of care for all and attending to the individual needs of some is a paradox of good nursing care (Grace, 2001). One the one hand, standards are necessary to ensure that all patients get the basics of what is needed, on the other hand, people have different contextual circumstances and needs that must be met to optimize their well‐being. This highlights the ethical tension between ensuring appropriate competencies to care for the population at hand and the ability to critically evaluate the ethical merits of standardized actions in particular care circumstances.

To address these imperatives, nursing curricula based on competence frameworks should keep historical nursing goals and perspectives as foregrounds. These are what unify the many settings and specialties of nursing and proposed competency domains. To guard against the reductionistic potential of competency‐based curricula (Jarvis‐Selinger et al., 2012; Krupat, 2018), an understanding of one's professional goals and responsibilities as well as what these entail in terms of questioning the status quo are critical. These permit assumptions that underlie proposed competencies, to be questioned and local or broader health policies that contribute to disparities confronted. Elsewhere, one of us has argued that nursing actions are inherently ethical in nature. This is because the profession exists to provide a critical human service not provided by others. As such nursing provides a ‘good’ and nurses can be critiqued to the extent that they do or do not focus sufficiently on delivering that ‘good’ in both everyday and conflictual situations (Grace, 2018). Thus, developing nurse moral agency is perhaps the most critical task of nursing education. The exercise of moral agency requires both an understanding of the ethical nature of nursing work and skills in ethical decision‐making, including how to articulate the nursing perspective in collaborative interdisciplinary work and knowledge of the tools and language of healthcare ethics.

As the nursing profession adopts curricula frameworks such as the CanMEDS, the CanMEDS in conjunction with EPA, or the one by Englander and colleagues, care needs to be taken that the profession's goals and perspectives are not subsumed under the interests of other stakeholders. Commentators from medicine have also cautioned about uncritical acceptance of such frameworks. Krupat (2018) echoing the concerns of others, notes the importance of “identity formation of young physicians” and the need to focus on “issues of interconnectedness and the broader meaning of being a physician” (p. 372). These are profound ethical questions for the professions, how to improve the education of clinicians without losing the soul and ultimate purposes of the profession. Besides the issue of professional formation, although each of these framework account to a certain degree for addressing ethical issues in practice, translation into actual practice settings remains challenging. Indeed, there are indications that the frameworks do not provide sufficient guidance and that clinicians struggle when confronted with ethically demanding situations in practice (Aoun et al., 2019; Bellini et al., 2019; Busche et al., 2016; Englander et al., 2013; Englander & Carraccio, 2018; Giuliani et al., 2020; Naidu et al., 2020).

In‐line with Chapman (1999), we worry that while “the competency‐based approach to nursing education is an indisputable reality… nursing competencies must not be allowed to control the curriculum” (p.129). What should control the curriculum are nursing goals and perspectives, then nurse‐specific competencies are instruments to prepare nurses who work to achieve nursing goals and contribute nursing perspectives in interdisciplinary work. While competency frameworks tend to look tidy, precise, and all‐encompassing, the paradox is that they do not account for the necessary characteristics of expertise, “they distort and understate the very things they are trying to represent” (Norris, 1991; p. 4).

In view of the long tradition, of integrating knowledge and epistemology from other reference sciences, recognition of the backgrounds of such adopted frameworks is absolutely essential. In particular, it is imperative that the scope and the limits of such frameworks are studied. At the same time, a complete understanding of the nursing core is indispensable. Only with such knowledge is it possible to determine the limits of frameworks from other disciplines that are adopted into nursing. Subsequently, the differences of the frameworks and, thus, the identification of gaps will lead to the formulation of additional measure to insure that nursing's core competencies as formulated by Hamric and Hanson (Tracy & O'Grady, 2019) for example can be addressed and fostered. In turn, the lessons learned may provide guidance to the medical profession and its struggle to implement the CanMEDS, or CanMEDS associated with EPA or the Englander and colleagues' model.

## 6 CONCLUSION

Shared competency frameworks may be helpful for fostering interprofessionality in healthcare; and interprofessional collaborations are often necessary for good patient care. However, unqualified acceptance of them is not warranted. We were interested to explore the capacity of competency frameworks to facilitate the development of moral agency and ethically sound nursing practice. However, we find the uncritical adoption both of competency frameworks and those where the foundations and inception are in disciplines other than nursing, to be problematic.

Our exploration resulted in some cautions. We urge curriculum planners to understand those competency frameworks may well offer structure but that the ultimate aim of the nursing profession is to develop nurses who can think outside of received views, provide ethically grounded service and care well. Competency frameworks are instruments to be wisely used and their limits understood. Detailed knowledge of the respective theoretical foundations and traditions of each profession is indispensable in determining the scope and limitations of such frameworks. Because medical competency frameworks are already being or about to be utilized in some countries, we recommend a careful ongoing review of how such frameworks account for: nurses' professional formation, their critical attitudes towards institutional, social, and the environmental impediments to good healthcare, the development of nurses' moral agency and confidence in ethical decision‐making by nurses. All of these are necessary for the nursing professions to ensure that the goals related to care of individuals and the provision of a societal good can be pursued and realized.

## CONFLICT OF INTEREST

The authors declare no conflict of interest.

## Data Availability

This manuscript is based on literature. The complete reference list is provided.
